# Altered expression of miR-181a and miR-146a does not change the expression of surface NCRs in human NK cells

**DOI:** 10.1038/srep41381

**Published:** 2017-02-01

**Authors:** Mona Rady, Carsten Watzl, Maren Claus, Ola Khorshid, Laila Mahran, Khaled Abou-Aisha

**Affiliations:** 1 Microbiology and Immunology Department, German University in Cairo (GUC), New Cairo, Egypt; 2 Immunology Department, Leibniz Research Center for Working Environment and Human Factors (IfADo), Dortmund, Germany; 3 Medical Oncology Department, National Cancer Institute (NCI), Cairo, Egypt; 4 Pharmacology and Toxicology Department, German University in Cairo (GUC), New Cairo, Egypt

## Abstract

MicroRNAs (miRNAs) play an important role in regulating gene expression and immune responses. Of interest, miR-181a and miR-146a are key players in regulating immune responses and are among the most abundant miRNAs expressed in NK cells. Bioinformatically, we predicted miR-181a to regulate the expression of the natural cytotoxicity receptor NCR2 by seeded interaction with the 3′-untranslated region (3′-UTR). Whereas, miR-146a expression was not significantly different (*P* = 0.7361), miR-181a expression was, on average 10-fold lower in NK cells from breast cancer patients compared to normal subjects; *P* < 0.0001. Surface expression of NCR2 was detected in NK cells from breast cancer patients (*P* = 0.0384). While cytokine receptor-induced NK cell activation triggered overexpression of miR-146a when stimulated with IL-2 (*P* = 0.0039), IL-15 (*P* = 0.0078), and IL-12/IL-18 (*P* = 0.0072), expression of miR-181a was not affected. Overexpression or knockdown of miR-181a or miR-146a in primary cultured human NK cells did not affect the level of expression of any of the three NCRs; NCR1, NCR2 or NCR3 or NK cell cytotoxicity. Expression of miR-181a and miR-146a did not correlate to the expression of the NCRs in NK cells from breast cancer patients or cytokine-stimulated NK cells from healthy subjects.

Breast cancer is the most common cancer diagnosed in women, accounting for 25.2% of all new cases in women^1^. Despite the development of more effective treatment options such as cytotoxic, hormonal, and anti-HER2 directed therapies for breast cancer, metastatic disease remains incurable, and one third of women with localized disease develop metastases and die of the disease^2^. Various immunotherapeutic strategies have been tested in preclinical and clinical settings for the treatment of breast cancer^3^. Natural killer (NK) cells play a critical role in the fight against tumour development and, therefore, hold great promise as an immunotherapeutic intervention for the treatment of breast cancer^3^,^4^,^5^. A more profound understanding of NK cell biology and function is, however, still needed for developing novel approaches for the effective therapeutic use of NK cells in cancer immunotherapy.

NK cells are innate immune lymphocytes important for early and effective immune responses against infections and cancer^6^. NK cells express a complex array of activating and inhibitory receptors that enable them to detect their cellular targets while sparing normal cells^7^,^8^,^9^. Activating receptors can detect internal changes that occur in damaged host tissues by the recognition of a wide range of ligands, whose expression is barely detectable in steady-state conditions but is induced by infection, transformation, or various forms of cellular stress^9^. Activating receptors include CD16, the C-type lectin-like receptors NKG2D, NKp80, and the heterodimer CD94/NKG2C, and the NCRs; NCR1 (also known as NKp46), NCR2 (also known as NKp44), and NCR3 (also known as NKp30)^7^,^10^. The NCRs are type I membrane proteins that belong to the immunoglobulin superfamily and are important for the stimulation of NK cell effector functions^11^,^12^,^13^. Whereas NCR1 and NCR3 are expressed both on resting and activated NK cells^12^,^13^,^14^, NCR2 is expressed on activated NK cells only^11^,^15^. NK cells regulate the level of their NCRs surface expression by a yet unclear mechanism.

miRNAs are a family of 21–25-nucleotide small, non-coding RNAs that negatively regulate gene expression at the post-transcriptional level^16^,^17^,^18^,^19^. miRNAs bind to their target mRNAs by imperfect complementarity^20^, usually at their 3′-UTRs and either promote their decay or inhibit their translation^21^,^22^. Bioinformatic analysis of the first known miRNA-regulated genes showed that pairing of miRNA nucleotides 2–8, called the seed region, to the 3′ UTR of the target mRNA is often important^23^,^24^. Several other binding modes have been reported as well^25^,^26^. Since their initial discovery, miRNAs have been continuously reported to be implicated in many cellular processes such as development, differentiation, proliferation, apoptosis, and malignant transformation^21^,^27^.

Recently miR-181a, a member of the miR-181 family, was shown to modulate T cell receptor (TCR) sensitivity and signalling strength by repressing multiple negative regulators in the TCR signalling pathway^28^,^29^. Expression of miR-181a was previously reported in both mouse^17^,^30^ and human^31^ NK cells. miR-181a is among the top 25 miRNAs expressed in resting and cytokine-stimulated human NK cells^31^. In mouse NK cells, miR-181a was 2-fold lower upon stimulation with IL-15^30^. Both miR-181a and miR-181b have been shown to directly regulate NK cell development by targeting a nemo-like kinase (NLK), which activates Notch signalling cascade^32^.

Among the first miRNAs shown to regulate the innate immune responses is miR-146a. Expression of miR-146a is increased following stimulation of toll-like receptors (TLRs) namely TLR-2, TLR-4 and TLR-5^33^,^34^,^35^. This TLR-mediated miR-146a expression is predominantly driven by the transcription factor nuclear factor-κB (NF-κB)^35^,^36^. miR-146a downregulates expression of IL-1 receptor associated kinases IRAK1 and IRAK2 as well as TNF receptor- associated factor 6 (TRAF6)^33^,^35^,^37^,^38^, which are key adaptor molecules downstream of TLR and interleukin-1 receptor signalling. Similar to miR-181a, expression of miR-146a was previously reported in both mouse^30^ and human^31^ NK cells. miR-146a is among the top 20 miRNAs expressed in resting and IL-15 activated mouse NK cells^30^. In human NK cells, miR-146a is upregulated by IL-15 stimulation^31^.

To our knowledge, there are no validated miRNAs regulating the expression of the NCRs. Preliminary examination of the 3′-UTR of NCR2 mRNA, however, shows the presence of a putative target site for the seed region of miR-181a. In the present study, we measured the expression of miR-181a and miR-146a in NK cells isolated from breast cancer patients and cytokine-stimulated NK cells isolated from healthy subjects. We also investigated the impact of altered expression of miR-181a and miR-146a on the surface expression of the NCRs and NK cell cytotoxicity.

## Results

### Prediction of miR-181a and miR-146a as a potential regulators of NCRs expression in NK cells

**Figure 1:**
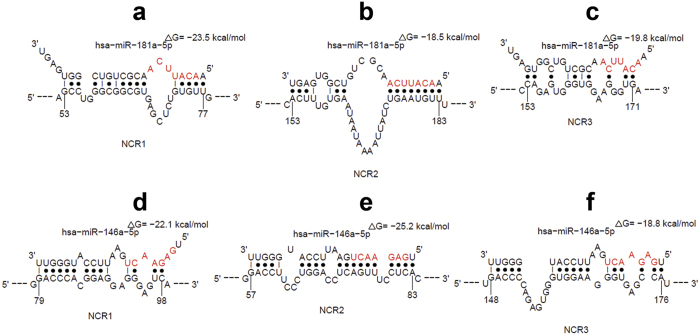
Bioinformatic prediction of miRNAs regulating NCRs expression. Prediction of miR-181a and miR-146a as potential regulators of the three NCRs using STarMiR software. Computational modelling showed the hybridisation of miR-181a and miR-146a and the 3′-UTRs of NCR1, NCR2 and NCR3 mRNAs. ΔG represents the calculated total energy change of the hybridisation. Possible conformations were generated by the Sfold web server available at http://sfold.wadsworth.org/cgi-bin/starmirtest2.pl.

Two different algorithms, TargetScan^24^ and miRWalk^39^ were used to demonstrate that the 3′-UTR of NCR2 mRNA contains a putative target site for the seed region of miR-181a (Supplementary Fig. S1). Moreover, the STarMiR software which predicts multiple potential miRNA:target mRNA bindings, first by predicting target secondary structures then by calculating the total energy change of the hybridisation^40^, predicted possible interaction of miR-181a and miR-146a with the 3′-UTRs of NCR1, NCR2 and NCR3 mRNAs (Fig. 1a–f). The aforementioned algorithms predicted that the binding between miR-181a and the 3′-UTRs of NCR1 and NCR3 mRNAs is “seedless” i.e., does not involve the seed region of the miRNA. Similarly, the interaction between the miR-146a and the 3′-UTRs of the three NCRs mRNAs was also “seedless”. However, the bindings appear to be thermodynamically favourable, as indicated by the negative total energy change due to the miRNA-mRNA hybridisation. Since the interaction between a miRNA and its target mRNA may lack perfect seed pairing but compensate by downstream complementarity at the 3′-end of the miRNA^25^,^26^, we decided to investigate the potential regulatory role of miR-181a and miR-146a on the expression of the three NCRs.

### Downregulation of miR-181a in NK cells freshly isolated from breast cancer patients

**Figure 2:**
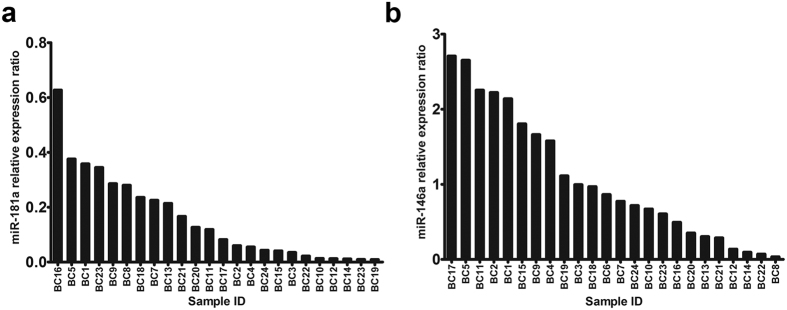
miR-181a and miR-146a relative expression in NK cells freshly isolated from breast cancer patients. Data are arranged in order of magnitude (n = 24). Relative expression ratio equals to 1 means the same expression level of miRNA in NK cells from breast cancer patient and healthy donors.

NK cells respond to circulating malignant tumour cells through dynamic engagement of multiple receptors resulting in NK cell activation. Evidence indicates that miRNA profiles of resting and activated NK cells are different^30^,^31^,^41^. Total RNA extracted from NK cells freshly isolated from breast cancer patients and healthy controls was used for miRNA expression analysis using RT-qPCR. Data were tested for normality using the D’Agostino-Pearson normality test and the *P* value for miR-181a relative expression was calculated using the non-parametric Wilcoxon signed-rank test. miR-181a expression was significantly lower in NK cells freshly isolated from breast cancer patients compared to healthy donors, *P* < 0.0001 (Fig. 2a). The minimum relative expression ratio was 0.03 (33-fold decrease), the maximum relative expression ratio was 0.6 (1.5-fold decrease), and the median was 0.1 (10-fold decrease). Similarly, the data for miR-146a were tested for normality using the D’Agostino-Pearson normality test and the parametric one sample *t* test was used to calculate the *P* value. miR-146a expression in NK cells freshly isolated from breast cancer patients and healthy donors were not significantly different, *P* = 0.7361 (Fig. 2b).

### NCR2 is expressed in NK cells from breast cancer patients

**Figure 3:**
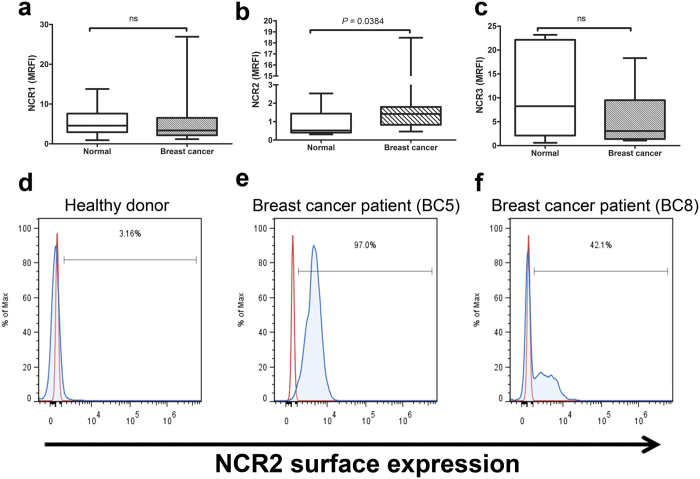
Analysis of NCRs expression on NK cells from breast cancer patients using freshly procured whole peripheral blood samples. Expression levels of NCR1 (**a**), NCR2 (**b**) and NCR3 (**c**) were analysed by flow cytometry from healthy donors (n = 6) (white boxes) and breast cancer patients (n = 21) (striped boxes). Median relative fluorescence intensity (MRFI) was calculated by subtracting median fluorescence intensity (MFI) of the unstained control from the MFI of the relevant monoclonal antibody divided by the MFI of the unstained control. Comparisons between healthy donors and patients were performed using the non-parametric Mann–Whitney U test. The lower boundary of the box indicates the 25^th^ percentile and the upper boundary the 75^th^ percentile. Bars above and below the box indicate the minimum and maximum data points. The line within the box marks the median. (**d**) Histogram representing an example of NCR2 expression on NK cells from a healthy donor. (**e**,**f**) Histograms representing examples of NCR2 expression on NK cells from two breast cancer patients (BC5 and BC8).

NCRs are important activating receptors for the antitumor activity of NK cells that are involved in the recognition and therefore killing of cancer cells. We analysed the expression of the NCRs, NCR1, NCR2, and NCR3 in NK cells from breast cancer patients and healthy donors using flow cytometry (Fig. 3). Cell surface analysis of NCRs was performed through 3-color flow cytometry using freshly procured whole peripheral blood samples after lysis of RBCs. NK cells were defined as CD3^−^CD56^+^ cells within the lymphocyte gate and the expression of NCRs was referred to this population. Supplementary Fig. S2 shows the flow cytometry gating strategy used to analyse the expression of the three NCRs using freshly procured whole peripheral blood samples. Expression of NCR1 (Fig. 3a) and NCR3 (Fig. 3c) were not significantly altered in breast cancer patients, *P* = 0.3562 and *P* = 0.6618, respectively. Expression of NCR2 was detected in NK cells from breast cancer patients compared to healthy donors, *P* = 0.0384 (Fig. 3b,d–f). Relative miR-181a and miR-146a expression in freshly isolated NK cells from breast cancer patients did not correlate to the level of surface expression of the three NCRs (Table 1).

### Cytokine stimulation of resting NK cells triggered overexpression of miR-146a

**Figure 4:**
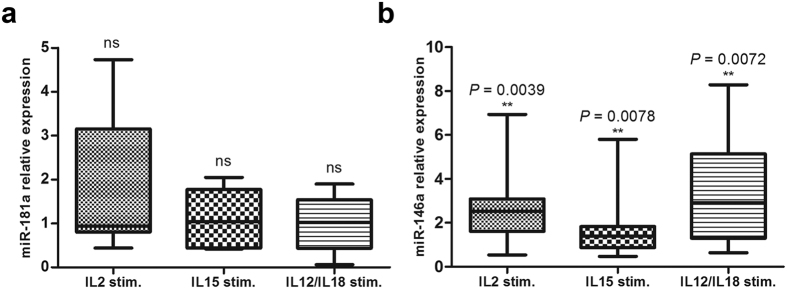
Effect of cytokine stimulation of resting NK cells on miR-181a and miR-146a expression. Relative miR-181a and miR-146a expression was quantified in NK cells isolated from healthy subjects and stimulated with IL-2, IL-15, or IL-12/IL-18 mixture. Data were tested for normality using the D’Agostino-Pearson normality test and the one sample *t* test or Wilcoxon signed-rank test were used when appropriate by testing if the mean or median is significantly different from 1. The lower boundary of the box indicates the 25^th^ percentile and the upper boundary the 75^th^ percentile. Bars above and below the box indicate the minimum and maximum data points. The line within the box marks the median. miRNA expression analysis in unstimulated and cytokine-stimulated NK cells (n = 9) was performed using RT-qPCR by normalizing the unstimulated NK cells to 1. U6 and U24 RNA were used as internal reference controls in all experiments.

To test the effects of IL-2, IL-15, IL-12/IL-18 stimulation of resting NK cells on miR-181a and miR-146a expression, NK cells freshly isolated from 9 healthy donors were stimulated with IL-2, IL-15, or IL-12/IL-18 for 48 hours. Cytokine stimulation did not affect the expression of miR-181a (IL-2 (*P* = 0.1111), IL-15 (*P* = 0.4768), or IL-12/IL-18 (*P* = 1.0000)) compared to the unstimulated NK cells (Fig. 4a). On the other hand, miR-146a expression was significantly higher in cytokine-stimulated NK cells (IL-2 (*P* = 0.0039), IL-15 (*P* = 0.0078), and IL-12/IL-18 mixture (*P* = 0.0072)) compared to the unstimulated NK cells (Fig. 4b). The relative expression of miR-181a or miR-146a in cytokine-stimulated NK cells did not correlate to the levels of surface expression of the three NCRs (Table 2).

### Overexpression or knockdown of miR-181a or miR-146a in primary cultured human NK cells isolated from healthy subjects did not affect the expression of the NCRs or NK cell cytotoxicity

**Figure 5:**
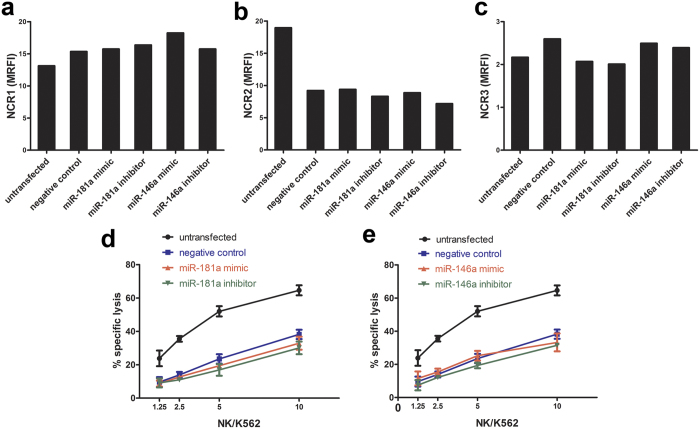
Impact of miR-181a and miR-146a overexpression and knockdown on expression of NCRs and NK cell cytotoxicity. Primary cultured human NK cells isolated from healthy subjects were transfected with miR-181a or miR-146a mimics and inhibitors to alter the expression of miR-181a and miR-146a. Surface analysis of NCRs (**a**–**c**) was performed through single-color flow cytometry. MRFI was calculated by subtracting the MFI of the isotype-matched control from the MFI of the relevant monoclonal antibody divided by the MFI of the isotype-matched control. Primary cultured human NK cells isolated from healthy subjects transfected with miR-181a or miR-146a mimics and inhibitors were used as effector cells in a ^51^Cr-release assay against K562 cells at different E:T ratios (**d,e**). Data represent means ± SD of triplicates. These are representative data of two independent experiments.

In order to manipulate the expression of miR-181a and miR-146a, primary cultured human NK cells isolated from healthy subjects were transfected with synthetic miRNA mimics or inhibitors. We confirmed successful delivery of genetic material into primary cultured NK cells isolated from healthy subjects by transfecting NK cells with 2 μg pmaxGFP™ Vector supplied with the P3 Primary Cell 4D-Nucleofector^®^ X Kit using the same procedures described for the transfection of miRNA mimics and inhibitors (Supplementary Fig. S3). Successful miRNA overexpression and knockdown were verified by RT-qPCR 72 hours after miRNA transfection. Compared to the negative control (NK cells transfected with single-stranded, modified RNA that has no homology to any known mammalian gene), miR-181a and miR-146a were successfully overexpressed after transfecting the miRNA mimics, with relative expressions of 16.397 for miR-181a and 78.380 for miR-146a. Also, compared to the negative control, successful knockdown of miR-181a and miR-146a was achieved by transfecting the miRNA inhibitors, with relative expressions of 0.047 for miR-181a and 0.121 for miR-146a. 72 hours post transfection (the time by which expressed NCRs prior to the transfection experiment would be turned over, Supplementary Fig. S4), expression of NCR1, NCR2 and NCR3 were measured using flow cytometry. Compared to the negative control, surface expression levels of NCR1, NCR2 or NCR3 were not affected by the altered expression of neither miR-181a nor miR-146a (Fig. 5a–c). Further, 72 hours post transfection, transfected NK cells were used as effector cells in a ^51^Cr-release assay against K562 cells. Also here, NK cell cytotoxicity did not appear to be affected by the altered expression of miR-181a or miR-146a (Fig. 5d,e).

## Discussion

NK cells are important mediators of antitumor immunity limiting their growth and dissemination. Evidence exists that the molecular program of resting and activated NK cells is quite distinct^42^. Moreover, previous studies demonstrated that the miRNA profiles of resting and activated NK cells are different^30^,^31^,^41^. In the present study, three algorithms predicted that the 3′-UTR of NCR2 mRNA contains a putative target site for the seed region of miR-181a. We analysed the steady state levels of miR-181a and miR-146a in NK cells isolated from healthy subjects and from breast cancer patients. Our analysis shows that the levels of miR-181a are, on average, 10-fold lower in NK cells freshly isolated from breast cancer patients. In a study on the role of miR-181a in the regulation of TCR receptor signalling in mouse T cells, enforced overexpression of miR-181a augmented TCR-mediated T cell activation by repressing multiple negative regulators in the TCR signalling pathway^28^. Although miR-181a expression did not change the TCR density on the cell surface, miR-181a quantitatively reduced the protein levels of multiple protein tyrosine phosphatases namely, SHP-2, PTPN22, DUSP5, and DUSP6^28^. Given that all of these phosphatases are expressed in NK cells^42^, we suggest that NK cells may utilize miR-181a to modulate signal strength downstream of receptor engagement to its ligand on cancer cells in breast cancer patients. Sprouty homolog 2 (Spry2), a member of the Sprouty family of transcriptional regulators, is another experimentally validated target for miR-181a^43^. Previously, Spry2, which is a negative regulator of the MAPK/ERK pathway^44^,^45^,^46^, was shown to have high and specific expression in NK cells^42^, suggesting that this protein might represent a regulator of the activation program of NK cells through inhibition of the MAPK/ERK pathway. Thus, the decreased expression of miR-181a in NK cells in breast cancer patients would help to fine-tune NK cell-mediated immune response to breast cancer by targeting Spry2.

Here, we also show that NCR2 is expressed on NK cells from breast cancer patients. In contrast to NCR1 and NCR3, NCR2 is not expressed on resting NK cells but only on activated NK cells^11^,^15^. This suggests that NK cells from breast cancer patient have an activated phenotype, possibly induced by the interaction with cancer cells. Knowledge of the molecular nature of tumour-associated NCR ligands in general and NCR2 specifically is still scarce. NCR2 can activate NK cells via its association with the immunoreceptor tyrosine-based activation motif (ITAM)-containing partner chain DAP12. However, NCR2 also contains an immunoreceptor tyrosine-based inhibitory motif (ITIM) in its own cytoplasmic tail that has been reported to be functional upon binding to the cellular ligand, proliferating cell nuclear antigen (PCNA)^47^. Although its expression was known to be restricted to the nucleus, PCNA is recruited by tumour cells to cell synapses with NK cells^47^. Before PCNA was described as a ligand for NCR2, it was already reported that its expression inhibits the killing of tumour target cells by NK cells^48^. Indeed, overexpression of recombinant PCNA resulted in a reduced IFN-γ secretion by IL-2-activated primary NK cells^47^. PCNA was previously reported to be highly expressed in primary breast tumours^49^. Recently, an activating cellular ligand for NCR2 was identified, NKp44L^50^. NKp44L is highly detected in several tumour cell lines and not detected in healthy tissues^50^. So far, there are no available expression data about NKp44L in primary breast tumours. Therefore, whether expression of NCR2 on NK cells from breast cancer patients would impact NK cell activation negatively or positively depends on the expression of inhibiting (PCNA) versus activating (NKp44L) cellular ligands on breast cancer cells.

Here we show that the surface expression of NCR1and NCR3 in NK cells from breast cancer patients, though lower than normal subjects, this difference was not statistically significant. Previously, reduced expression of NCRs has been shown to be associated with some forms of cancers. Expression of NCR1 was previously reported to be reduced in acute myeloid leukaemia^51^,^52^ and cervical cancer^53^. Similar to our results, a previous study reported no significant difference in expression of NCR1 in breast cancer compared to healthy subjects^54^. Expression of NCR3 was found to be reduced in acute myeloid leukaemia in one study^51^, while another study reported no significant difference in NCR3 expression in acute myeloid leukaemia compared to healthy subjects^52^. In breast^54^ and cervical cancers^53^, NCR3 expression was reported to be significantly reduced compared to healthy donors.

We also show that cytokine receptor-induced NK cell activation triggers overexpression of miR-146a. This was observed by stimulating freshly isolated NK cells with IL-2, IL-15, or IL-12 and IL-18 mixture. It is now evident that the interaction of cytokines with their receptors leads to the activation of multiple signalling pathways eliciting different biological responses in NK cells. Cytokine receptors such as IL-2 and IL-15 receptors belong to the IL-2 receptor family that signals through a common γ-chain and associate with either Jak1 or Jak3 kinases. IL-12–type cytokine receptors, however, associate with either Jak2 or Tyk2 kinases. Cytokine interaction with its specific receptor leads to the activation of these Jak kinases and subsequent activation of the PI3/AKT signalling pathway^55^,^56^, which has been suggested to eventually trigger the activation of NF-κB^57^,^58^,^59^. IL-18 receptor associates with MyD88 and transmits its signal by triggering the MyD88-dependent IL-1 receptor signalling pathway. Upon cytokine stimulation of IL-18 receptor, MyD88 recruits IRAK4 and the MyD88-IRAK4 complex recruits IRAK2 or IRAK1. This Myddosome complex then phosphorylates and activates TRAF6 eventually activating NF-κB. Being an NF-κB-dependent gene^35^,^36^, activation of NF-κB following cytokine stimulation of resting NK cells explains the induced overexpression of miR-146a.

Experimental approaches for miRNA target determination in specific cell types, although challenging, are indispensable to confirm the biologically relevant targets of a given miRNA^60^,^61^. Our approach to investigate the impact of altered expression of miR-181a and miR-146a on surface NCRs expression was to transfect primary cultured human NK cells isolated from healthy subjects with miRNA mimics and inhibitors followed by downstream measurement of the level of surface expression of NCRs using flow cytometry. miRNA mimics have the same functional sequence as the natural mature miRNA and therefore mimic endogenous miRNA activity. miRNA inhibitors, on the other hand block endogenous miRNA activity and therefore, halt miRNA-induced gene silencing. Although this is a direct and straight forward approach to test the ultimate effect of miR-181a and miR-146a dysregulation on the protein levels of NCRs, this approach probably suffers from some limitations. First, it was not possible to validate the bioinformatically predicted miRNA:mRNA binding. Although a reporter gene assay could test whether a given miRNA binds a specific RNA target sequence or not, it does not provide evidence that this interaction occurs endogenously. Second, despite successful miRNA overexpression and knockdown as evidenced by measuring the level of mature miR-181a and miR-146a in NK cells post nucleofection, we have not tested previously validated targets particularly for miR-181a^28^ which was bioinformatically predicted to interact canonically with its seed region to the 3′-UTR of NCR2 mRNA. Previously, it was demonstrated that T lymphocyte activation is associated with 3′-UTR shortening^62^. This 3′-UTRs shortening is due to alternative polyadenylation that leads to mRNAs with varying length of their 3′-UTRs. Thus, the same gene that is subject to miRNA-mediated regulation when it has a long 3′-UTR is less likely to be targeted by miRNAs once it shortens its 3′-UTR^63^. In the present study, for overexpression and knockdown of miR-181a and miR-146a we used primary cultured human NK cells isolated from healthy subjects that were subjected to different cytokines and growth factors following their isolation to maintain them in cultures. Therefore, a deeper insight into alternative polyadenylation during NK cell activation and proliferation would be needed to judge on possible differential regulation of short versus long 3′-UTRs of NCRs mRNAs by miRNAs. An alternative to using primary cultured human NK cells is using freshly isolated NK cells from healthy donors for the miRNA overexpression and knockdown experiments. Besides the fact that using freshly isolated NK cells for nucleofection experiments is technically challenging, the number of NK cells needed for conducting these experiments adds another technical challenge.

Individual miRNAs can be redundant, that is multiple related miRNA family members can act on a similar set of target genes^63^. For example, the other members of the miR-181 family miR-181b, miR-181c, and miR-181d were also “predicted” by TargetScan software and miRWalk database to regulate NCR2 (Supplementary Fig. S1). Thus, the impact of altering the endogenous level of a single miRNA using miRNA mimics and inhibitors, for example, might be compensated by another miRNA. Moreover, miRNAs generally regulate their target genes with often less than 50% repression^64^,^65^. Therefore, separating real regulation from technical artefacts or biologic fluctuation is challenging^63^. The situation is further complicated by the recently suggested “two-way” regulation; the availability of a given target regulates the levels of its cognate miRNA regulator^25^.

The other strategy we employed here was to measure the expression of miR-181a and miR-146a in NK cells freshly isolated from breast cancer patients (Table 1) as well as in cytokine-stimulated NK cells isolated from healthy subjects (Table 2) in correlation to surface NCRs expression. Taken together our results demonstrate that altered expression of miR-181a and miR-146a whether experimentally induced or as observed in breast cancer patients or cytokine-stimulated NK cells isolated from healthy subjects does not change the surface expression of any of the three NCRs.

In conclusion, miRNA expression analysis revealed that miR-181a is downregulated in NK cells from breast cancer patients compared to healthy subjects. Flow cytometric analysis of NCRs revealed that NCR2 is expressed on NK cells from breast cancer patients possibly induced by the interaction with cancer cells. Moreover, cytokine stimulation of resting NK cells triggered overexpression of miR-146a, but not miR-181a. Although miR-181a was predicted to interact canonically with its seed region to the 3′-UTR of NCR2 mRNA, altered expression of miR-181a alone did not affect the surface expression of NCR2 in human NK cells.

## Methods

### Bioinformatics

Sequences of mature miRNAs were obtained from the miRNA Registry at miRBase (http://www.mirbase.org) as shown in Supplementary Table S1. NCRs’ sequences were obtained from National Center for Biotechnology Information Nucleotide database (http://www.ncbi.nlm.nih.gov/) as shown in Supplementary Table S2. The prediction algorithms TargetScan human^24^, miRWalk^39^, and STarMiR^40^ were used to search for NCR genes that can be targeted by miR-181a or miR-146a.

### Peripheral blood samples

All experimental procedures were conducted in accordance with the relevant local regulations and guidelines of the National Cancer Institute (NCI), Cairo University, the German University in Cairo and the Leibniz Research Centre for Working Environment and Human Factor, Germany, and conformed to the Declaration of Helsinki. The use of all human tissue was approved by the local ethical committees of the National Cancer Institute (NCI), Cairo University, the German University in Cairo and the Leibniz Research Centre for Working Environment and Human Factor, Germany, and informed consents were obtained in accordance with the Declaration of Helsinki. Peripheral blood was sampled from patients admitted to the NCI-Cairo, with histologically confirmed diagnosis of primary breast cancer. Peripheral blood was also procured from healthy donors. Clinicopathological characteristics of the breast cancer patients are shown in Table 3. Exclusion criteria for both patients and controls included any concomitant disease state, which may cause chronic inflammation.

### Isolation of NK cells

Peripheral blood of patients with breast cancer and healthy donors was directly obtained in heparin vacutainers, BD Biosciences, San Jose, CA, USA. Peripheral blood mononuclear cells (PBMCs) were isolated from whole blood using lymphocyte separation medium, Lonza Walkersville, Inc. Houston TX, USA. The cell pellet was washed twice in calcium- and magnesium-free Dulbecco’s PBS-2% FCS-2 mM EDTA, enumerated on a haemocytometer with trypan blue and resuspended at 5 × 10^7^/500 μl. NK cells were isolated through immunomagnetic negative selection using Dynabeads^®^ Untouched™ Human NK Cells kit, Invitrogen Cergy-Pontoise, France, following the manufacturer’s recommendations. The purity (% of CD3^−^CD56^+^) of NK cells measured by flow cytometry confirmed that these cells were more than 98% CD3^−^CD56^+^ NK cells and less than 2% CD3^+^.

### Transfection of primary human NK cells

Primary NK cells from healthy subjects were transfected after 10–14 days of culture in vericyte^®^ NK cell growth medium, Medicyte, Heidelberg, Germany using the nucleofection method (Lonza). The following synthetic miRNA mimics and inhibitors (Qiagen, Hilden, Germany) were used: Syn-hsa-miR-181a-5p (catalogue # MSY0000256), Syn-hsa-miR-146a-5p (catalogue # MSY0000449), Anti-hsa-miR-181a-5p (catalogue # MIN0000256) and Anti-hsa-miR-146a-5p (catalogue # MIN0000449), in addition to miScript Inhibitor Negative control (catalogue # 1027271). 2–3 × 10^6^ cells per sample were transfected with 300 pmol of synthetic miRNA in 100 μl nucleofector solution of P3 Primary Cell 4D-Nucleofector^®^ X Kit (catalogue # V4XP-3024), Lonza, Walkersville, Inc. Houston TX, USA and the nucleofection program DK100 using the 4D-Nucleofector™ System, Lonza, following the manufacturer’s instructions at all other steps. Immediately post nucleofection, cells were cultured in IMDM-10% human serum-1% Pen/Strep-1% sodium puruvate-1% NEAA medium and incubated in a humidified incubator at 37 °C and 5% CO_2_ for 72 hours.

### Cytokine stimulation of resting NK cells

1 × 10^6^ NK cells isolated from 9 healthy donors were cultured in 1 ml IMDM-10% human serum-1% Pen/Strep-1% sodium puruvate-1% NEAA without and with the addition of 100 IU/ml of IL-2, 5 ng/ml of IL-15 or 5 ng/ml of IL-12 and 20 ng/ml of IL-18 mixture (all purchased from PanBiotech, Aidenbach, Germany) for 48 hours. Unstimulated and stimulated NK cells were harvested for miR-181a and miR-146a expression analysis by RT-qPCR and NCRs expression analysis by flow cytometry.

### Total RNA isolation

NK cells were lysed in 1 ml TRIzol reagent, Invitrogen Cergy-Pontoise, France and total RNA was extracted from NK cell lysates using TRIzol reagent following the manufacturer’s instructions. RNA concentration and purity were measured using a NanoDrop 2000 UV-Vis spectrophotometer, Thermo Scientific, UK. The 260/280 ratio were between 1.7 and 2.1 for all samples. RNA integrity was assessed by running total RNA on a denaturing 15% polyacrylamide gel. Total RNA had clearly visible tRNA, 5 S rRNA, and 5.8 S rRNA bands for all samples.

### RT-qPCR

miRNA expression analyses were performed using the TaqMan MicroRNA Reverse Transcription Kit, TaqMan Universal PCR Master Mix, and TaqMan microRNA Assay primers for human miRNAs (Applied Biosystems, Warrington, UK); miR-181a (assay ID 000480) and miR-146a (assay ID 000468) as well as the internal controls; RNU6B (assay ID 001093) and RNU24 (assay ID 001001). Reverse transcription reactions contained 10-ng total RNA samples, 1 mM of dNTPs, 1 × RT primer, 1 × RT buffer, 0.25 U of RNAse inhibitor, and 3.3 U of reverse transcriptase in 15-μl reaction volume. The 15-μl reactions were incubated for 30 minutes at 16 °C, 30 minutes at 42 °C, 5 minutes at 85 °C, and then held at 4 °C in a thermal cycler (Biorad). QPCR was performed using MX3005 P™ quantitative real-time PCR system, Stratagene, La Jolla, San Diego, California, USA. QPCR reactions contained 1.33 μl reverse transcription product, 1 × PCR Master mix, and 1 × TaqMan-primers mix in a 20-μl reaction volume. Reactions were incubated in a 96-well plate sealed with optical adhesive PCR film, Eppendorf AG, Hamburg, Germany at 50 °C for 2 minutes, 95 °C for 10 minutes, and followed by 40 cycles of 95 °C for 15 seconds and 60 °C for 1 minute. All reactions were run in duplicates. The data analysis was performed with MxPro QPCR software version 4.01, Stratagene, La Jolla, San Diego, California, USA. The relative expression of mature miRNA (miR-181a and miR-146a) was calculated using the comparative CT method^66^ after normalization to the average expression of RNU6B and RNU24 yielding a ΔCt value. The −ΔΔCt value was then calculated by subtracting the average ΔCt value of 5 NK cell samples from healthy donors from the respective ΔCt values of NK cell samples from cancer patients. The −ΔΔCt values were then used to calculate the relative miRNA expression ratios (2^−ΔΔCt^).

### Flow cytometry

Cell surface analysis of NCRs in NK cells from breast cancer patients and healthy controls was performed through 3-color flow cytometry using Attune^®^ Acoustic Focusing Cytometer and Attune^®^ software pack v1.2.5 (Applied Biosystems, Foster City, CA, USA). 100 μl of fresh whole blood were treated with CAL-LYSE^TM^ Lysing Solution, Invitrogen, Cergy-Pontoise, France to lyse RBCs. Cells were immunostained by incubation with the appropriated antibodies for 30 minutes in the dark at 4 °C. The following mouse monoclonal antibodies were used for each sample: fluorescein isothiocyanate (FITC)–conjugated anti-CD3 and phycoerythrin-Cy5.5 (PE- Cy^®^ 5.5)–conjugated anti-CD56 antibodies, both purchased from Invitrogen, Cergy-Pontoise, France, in combination with phycoerythrin (PE)–conjugated anti-NCR1, anti-NCR2, or anti-NCR3 all purchased from R&D Systems, Bad Nauheim, Germany. NK cells were defined as CD3^−^CD56^+^ cells within the lymphocyte gate and the expression of NCRs was referred to this population. For freshly isolated, primary cultured, or transfected human NK cells, surface analysis of CD3, CD56, and NCRs was performed through single-color flow cytometry with the use of BD FACSCalibur flow cytometer. About 1 × 10^5^ NK cells were immunostained by incubation with the appropriated antibodies for 30 minutes in the dark at 4 °C. The following PE-conjugated mouse monoclonal antibodies were used: anti-CD3, anti-CD56, anti-NCR1, anti-NCR2, and anti-NCR3 all purchased from Biolegend, San Diego, CA, USA. Results were evaluated with the FlowJo sofware available from TreeStar, Ashland, OR.

### ^51^Cr release assay

72 hours post nucleofection, NK cell cytotoxicity was measured using ^51^Cr release assay as described previously^67^. Briefly, target cells (K562 cells) were grown to mid-log phase and 5 × 10^5^ cells were labelled in 100 μl CTL medium (IMDM medium supplemented with 10% FCS, and 1% Pen/Strep) with 100 μCi ^51^Cr for 1 h at 37 °C. Cells were washed twice in CTL medium and resuspended in CTL medium at 5 × 10^4^ cells/ml. Effector cells were mixed at different effector to target (E:T) ratios with 5000 labelled target cells/well in a 96-well V-bottom plate and incubated for 4 hours at 37 °C. Maximum release was determined by incubation in 2% Triton X-100. For spontaneous release, targets were incubated without effectors in CTL medium alone. All samples were done in triplicate. Supernatants were harvested and ^51^Cr release was measured in a PerkinElmer gamma counter. Percent specific release was calculated as ([experimental release-spontaneous release]/[maximum release-spontaneous release]) × 100. The ratio between maximum and spontaneous release was at least 4 in all experiments.

### Statistical analysis

Statistical analysis and graphical presentations were performed with GraphPad Prism5.0 (GraphPad). Data were tested for normality using the D’Agostino-Pearson normality test. Extreme outliers were tested using the Grubb’s test at *P* = 0.01, as described in the International Standard Organization document ISO 5725-2^68^. The parametric one sample *t* test, the non-parametric Wilcoxon signed-rank test, Mann–Whitney U test, and Spearman’s rank correlation coefficient (r) were used when appropriate, with *P* < 0.05 considered significant: *P < 0.05, **P < 0.01, ***P < 0.001.

## Additional Information

**How to cite this article:** Rady, M. *et al*. Altered expression of miR-181a and miR-146a does not change the expression of surface NCRs in human NK cells. *Sci. Rep.* **7**, 41381; doi: 10.1038/srep41381 (2017).

**Publisher's note:** Springer Nature remains neutral with regard to jurisdictional claims in published maps and institutional affiliations.

## Supplementary Material

Supplementary Information

## Figures and Tables

**Table 1:** Summary of Spearman’s rank correlation coefficient (r) for statistical analysis of the correlation between the levels of miR-181a and miR-146a and NCRs’ surface expression on NK cells from breast cancer patients using freshly procured whole peripheral blood samples.

	miR-181a relative expression			miR-146a relative expression		
	NCR1 (MRFI)	NCR2 (MRFI)	NCR3 (MRFI)	NCR1 (MRFI)	NCR2 (MRFI)	NCR3 (MRFI)
r	−0.2143	0.2857	−0.08442	−0.05584	−0.03117	−0.02727
*P* value (two-tailed)	0.3509	0.2093	0.7160	0.8100	0.8933	0.9066

**Table 2:** Summary of Spearman’s rank correlation coefficient (r) for statistical analysis of the correlation between the levels of miR-181a and miR-146a and NCRs’ surface expression on cytokine-stimulated NK cells isolated from healthy donors.

Cytokine stimulation		miR-181a relative expression			miR-146a relative expression		
		NCR1 (MRFI)	NCR2 (MRFI)	NCR3 (MRFI)	NCR1 (MRFI)	NCR2 (MRFI)	NCR3 (MRFI)
**IL-2**	**r**	0.0000	0.09524	−0.3810	0.2143	0.7857	0.3333
	***P*** **value (two-tailed)**	1.0232	0.8401	0.3599	0.6191	0.0279	0.4279
**IL-15**	**r**	0.4762	0.5000	0.3333	0.2857	0.6905	0.4762
	***P*** **value (two-tailed)**	0.2431	0.2162	0.4279	0.5008	0.0694	0.2431
**IL-12/IL-18**	**r**	0.2143	0.5238	0.3095	0.5476	0.6190	0.5238
	***P*** **value (two-tailed)**	0.6191	0.1966	0.4618	0.1710	0.1150	0.1966

**Table 3:** Clinicopathological characteristics of breast cancer patients used in this study.

Group characteristic	Number
Healthy group	6
Breast cancer group	24
Invasive ductal carcinoma histological grade	
** I**	1
** II**	17
** III**	2
Lymph node metastases	
** Positive**	14
** Negative**	5
Oestrogen receptor (ER) status	
** Positive**	11
** Negative**	8
Progesterone receptor (PR) status	
** Positive**	10
** Negative**	9
HER2/neu status	
** Positive**	9
** Negative**	10
Menopausal status	
** Premenopausal patients**	14
** Postmenopausal patients**	8

Data not known for 4 cases of histological grade, 5 cases of lymph node metastasis, 5 cases each for ER, PR and HER2/neu status, and 2 cases of menopausal status.
