# Supplementary material for: Altered expression of miR-181a and miR-146a does not change the expression of surface NCRs in human NK cells
Source: Sci Rep. 2017 Feb 1;7:41381. doi: 10.1038/srep41381 (PMC5286401; doi:10.1038/srep41381)

Supplementary information

Title: Altered expression of miR-181a and miR-146a does not change the expression of surface NCRs in human NK cells

Authors: Mona Rady, Carsten Watzl, Maren Claus, Ola Khorshid, Laila Mahran, Khaled Abou-Aisha

Supplementary Table S1. miRNA information as obtained from the miRNA Registry at miRBase.

miRNA ID	Accession number	Previous IDs	Sequence
hsa-miR-181a-5p	MIMAT0000256	hsa-miR-181a	5'-aacauucaacgcugucggugagu-3'
hsa-miR-146a-5p	MIMAT0000449	hsa-miR-146; hsa-miR-146a	5'-ugagaacugaauuccaugggguu-3'

Supplementary Table S2. NCRs' mRNA information as obtained from the NCBI database.

NCR	NCBI name/gene ID	Accession number
NCR1	NCR1	NM_004829
NCR2	NCR2	NM_001199510.1
NCR3	NCR3	NM_147130

Supplementary Figure S1. Bioinformatic prediction of miR-181a regulating NCR2 expression. (a) Prediction of miR-181a by TargetScan software as a potential miRNA regulating the expression of NCR2. Results were generated using the web server of TargetScan available at http://www.targetscan.org/. (b) Prediction of miR-181a by miRWalk database as a potential miRNA regulating the expression of NCR2. Results were generated using the web server of miRWalk available at http://www.umm.uni-heidelberg.de/apps/zmf/mirwalk/.

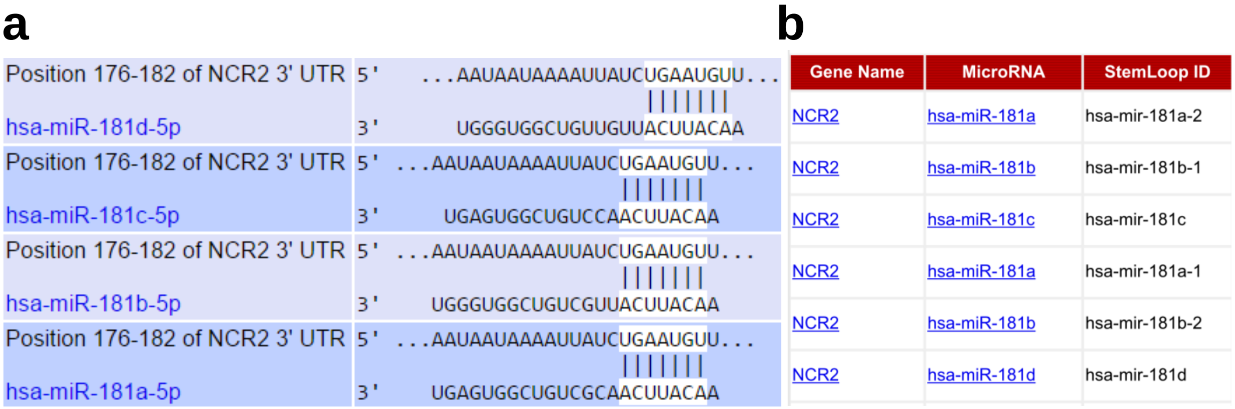

Supplementary Figure S2. Flow cytometry gating strategy to analyze NCRs expression on NK cells from freshly procured whole peripheral blood samples. Gating on peripheral blood lymphocytes using forward scatter (FSC) versus side scatter (SSC) parameters (left panel), NK cells were selected as CD3^{-}CD56^{+} cells within the lymphocyte gate (middle panel) and the expression of NK cell receptors referred to the CD3^{-}CD56^{+} region (right panel). In this example histogram shows the expression of NCR3 on NK cells.

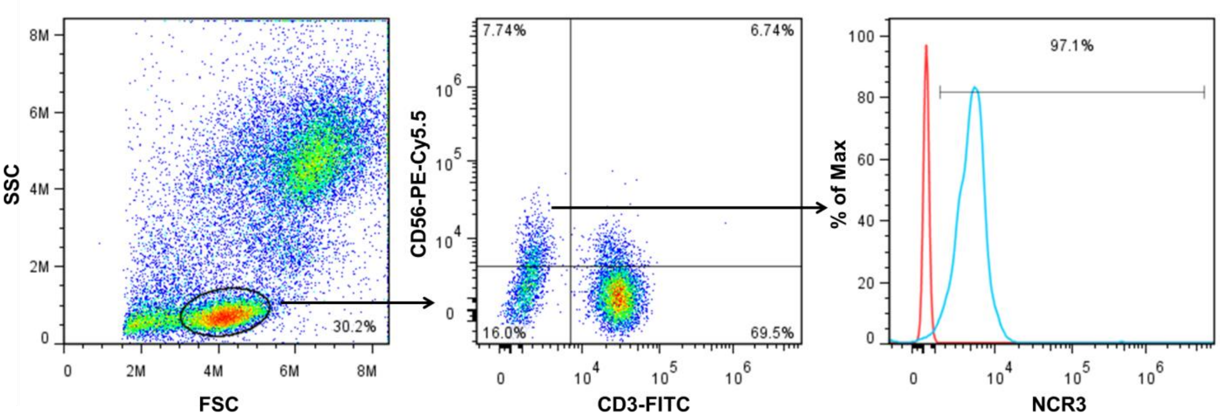

Supplementary Figure S3. FACS analysis of primary cultured human NK cells transfected with pmaxGFP™ Vector supplied with the P3 Primary Cell 4D-Nucleofector® X Kit. Within the viable lymphocyte gate 66% of NK cells were successfully transfected with GFP plasmid.

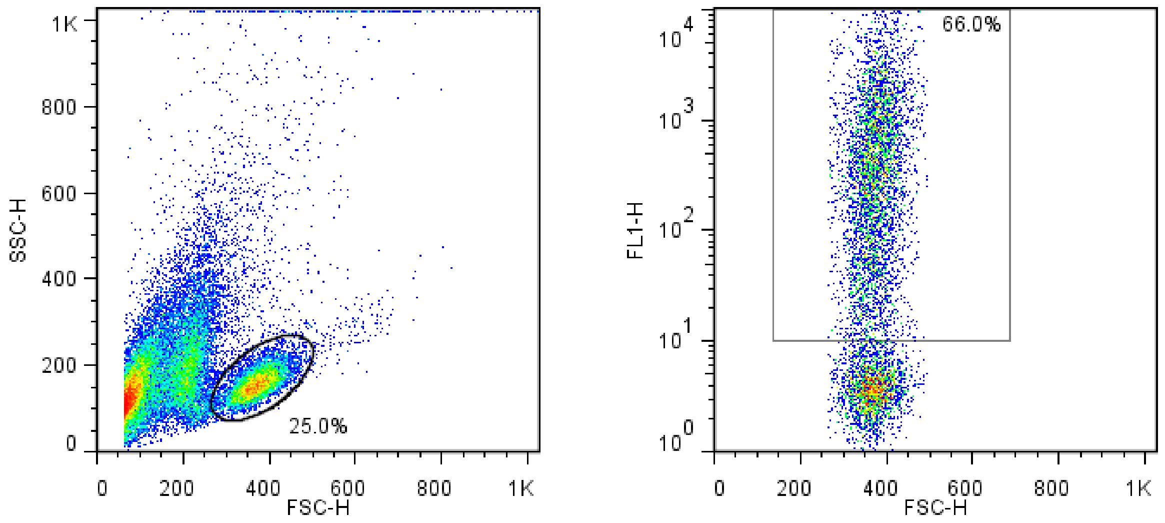

Supplementary Figure S4. Determination of rate of turnover of NCR1, NCR2, and NCR3 in primary cultured NK cells isolated from healthy subjects. NK cells were harvested 24 hrs, 48 hrs, and 72 hrs post cycloheximide (Sigma-Aldrich) treatment at a final concentration of 50 µg/ml in IMDM-10 % human serum-1 % Pen/Stryp-1 % sodium puruvate-1 % NEAA medium without cytokines (the same medium used to culture NK cells post nucleofection experiments). Cycloheximide is a glutarimide antibiotic derived from streptomycin-producing strains of *Streptomyces griseus* that inhibits eukaryotic protein synthesis. Surface expression of NCR1, NCR2, and NCR3 was analysed using flow cytometry. It required NCR1, NCR2, and NCR3 at least 72 hrs to be turned over.

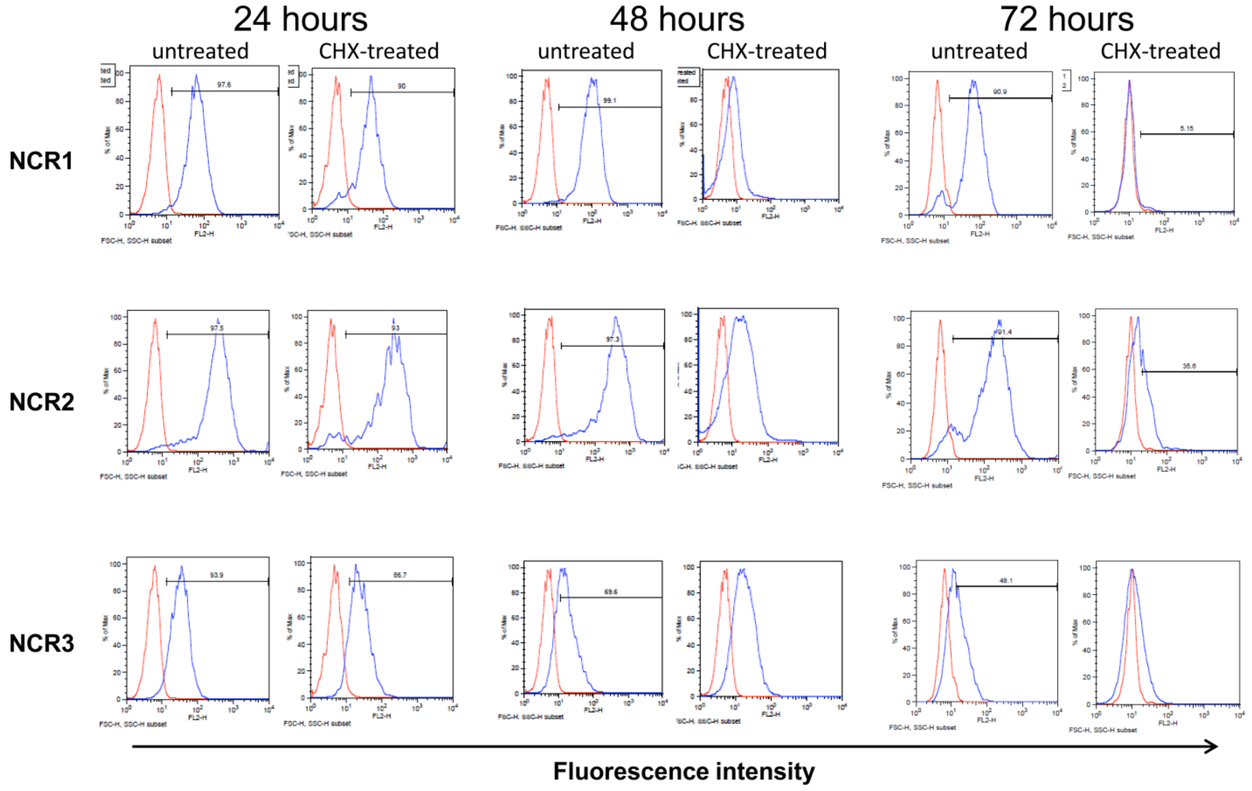

Supplement: Supplementary Information [file srep41381-s1.pdf]
